# An altered B cell compartment distinguishes low antibody responders among people with HIV

**DOI:** 10.64898/2026.09.18.26363407

**Published:** 2026-09-23

**Authors:** Karli R. Redinger, Samuel B. Warner, Armaan Jamal, Nuria Gallego Marquez, Matthew L. Mendoza, David E. Sanin, Michael J. Peluso, Alan L. Landay, Andrea L. Cox, Annukka A. R. Antar, Elizabeth A. Thompson

## Abstract

Chronic HIV infection can leave persistent immune dysregulation despite effective antiretroviral therapy (ART), but how this altered immune landscape affects antibody induction and durability after antigen exposure remains unclear. We used SARS-CoV-2 infection and vaccination as a model of humoral immunity to a novel antigen, evaluating anti-RBD IgG titers and immune cell phenotypes in people with HIV (PWH) and without HIV (PWoH) with hybrid immunity, stratified by early (<90 days) or late (≥90 days) post-exposure time points. PWH who failed to mount protective anti-RBD IgG titers early had a B cell compartment that was distinct from PWoH and high-titer PWH. Specifically, low-titer PWH had the highest expansion of activated naïve, CD11c⁺ age-associated, and IgM-skewed B cells with reduced regulatory marker expression, even after controlling for age, sex, BMI and days since infection. PWH who had low-titer responses during the late phase of the response had persistent CD11c-associated features with expansion of double-negative and altered class-switched memory B cell compartments. These B cell features correlated with CD4 and CD8 T cell activation and senescence-associated phenotypes, suggesting that failure to mount high titer antibody response and maintenance in a subset of PWH is shaped by coordinated immune dysregulation.

## Full Text Availability

The license terms selected by the author(s) for this preprint version do not permit archiving in PMC. The full text is available from the preprint server.

